# In silico analysis suggests the RNAi-enhancing antibiotic enoxacin as a potential inhibitor of SARS-CoV-2 infection

**DOI:** 10.1038/s41598-021-89605-6

**Published:** 2021-05-13

**Authors:** Amirhossein Ahmadi, Sharif Moradi

**Affiliations:** 1 Department of Biological Science and Technology, Faculty of Nano and Bio Science and Technology, Persian Gulf University, Bushehr, 75169, Iran; 2 Department of Stem Cells and Developmental Biology, Cell Science Research Center, Royan Institute for Stem Cell Biology and Technology, ACECR, Tehran, Iran

**Keywords:** Data integration, Data processing, Gene ontology, Gene regulatory networks, Viral infection, Molecular biology

## Abstract

COVID-19 has currently become the biggest challenge in the world. There is still no specific medicine for COVID-19, which leaves a critical gap for the identification of new drug candidates for the disease. Recent studies have reported that the small-molecule enoxacin exerts an antiviral activity by enhancing the RNAi pathway. The aim of this study is to analyze if enoxacin can exert anti-SARS-CoV-2 effects. We exploit multiple computational tools and databases to examine (i) whether the RNAi mechanism, as the target pathway of enoxacin, could act on the SARS-CoV-2 genome, and (ii) microRNAs induced by enoxacin might directly silence viral components as well as the host cell proteins mediating the viral entry and replication. We find that the RNA genome of SARS-CoV-2 might be a suitable substrate for DICER activity. We also highlight several enoxacin-enhanced microRNAs which could target SARS-CoV-2 components, pro-inflammatory cytokines, host cell components facilitating viral replication, and transcription factors enriched in lung stem cells, thereby promoting their differentiation and lung regeneration. Finally, our analyses identify several enoxacin-targeted regulatory modules that were critically associated with exacerbation of the SARS-CoV-2 infection. Overall, our analysis suggests that enoxacin could be a promising candidate for COVID-19 treatment through enhancing the RNAi pathway.

## Introduction

Since the emergence of SARS-CoV-2 virus in November 2019, COVID-19 has become the biggest challenge in the world^1^. Although several efforts are currently underway to develop COVID-19 vaccines, there is an urgent need to find new, effective treatments in order to decrease the mortality rate especially in regions and countries with the highest number of COVID-19 cases and deaths^2,3^. Various strategies including blocking viral entry into the host cells^4–6^, inhibiting viral replication^7,8^ and reducing cytokine storms^9,10^ have been proposed to relieve patients from COVID-19 symptoms. Based on these strategies, hundreds of clinical trials have been conducted and only a few drugs have been shown to slightly shorten the time to recovery or weakly reduce the rate of death among hospitalized patients^8,11^. However, there is still no specific efficacious medicine for COVID-19 that clearly reduces the death rate, making it critically inevitable to look for, and evaluate, new drug candidates for the effective treatment of COVID-19. As a potent antiviral strategy, the innate immune system could be exploited to fight the deadly infection caused by SARS-CoV-2.

The innate immune system, which functions as the first line of defense against viruses in the majority of mammalian cells, consists of the interferon (IFN) and the RNA interference (RNAi) pathways as major immune mechanisms against various viruses^12^. The IFN pathway is activated by viral components, thereby transcriptionally activating a large number of the so-called IFN-stimulated genes (ISGs)^13^. The activation of ISGs induces the production and secretion of various cytokines and chemokines which in turn recruit a large number of immune cells to the site of infection^14^. This pathway appears to be more active in mature cells (*i.e.* less active in stem and progenitor cells which are frequently infected by many viruses)^15^. Moreover, it can lead to a potentially life-threatening immune reaction called the cytokine release syndrome or cytokine storm which is resulted from an exaggerated immune response (i.e. a hyperactive IFN-mediated response) to the viral infection^16^. This immune overreaction is injurious to the host cells and might be induced by the SARS-CoV-2 infection^17^. In contrast, the RNAi pathway is an IFN-independent process of fighting viruses (therefore, does not induce a cytokine storm) and is typically more active in embryonic and non-mature (e.g. stem and progenitor) cells^18^. It involves the efficient degradation of the large viral RNAs which form secondary double-stranded structures thereby serving as substrates of the RNAi pathway^18^.

Fluoroquinolones such as enoxacin are broad-spectrum synthetic antibiotics used in different clinical conditions like urinary tract-, respiratory-, and systemic infections^19,20^. Although quinolones are known to typically inhibit DNA replication by targeting bacterial DNA gyrases^21^, a growing body of evidence has revealed that some members of this family of antibiotics could also inhibit viral helicases, attenuate cytokine production and pro-inflammatory reactions^22^, and more importantly enhance the RNAi process as an inflammation-free innate immune defense against viral infections^23^.

RNAi acts as a sequence-specific gene silencing process in which double-stranded RNAs (dsRNAs) such as short hairpin RNAs (shRNAs), viral RNAs, and microRNA (miRNA) precursors are cleaved by the RNase enzyme DICER to yield small interfering RNA (siRNA) duplexes. One strand of these duplexes is then preferentially incorporated into the so-called RNA‐induced silencing complex (RISC) to target complementary transcripts through Watson–Crick base‐pairing interactions^24^. The initiating dsRNAs can be either exogenous (*e.g.* viral RNAs or shRNAs) or endogenous (e.g. pre-miRNA transcripts) which are processed to generate siRNAs and miRNAs^25,26^. miRNAs are short non-coding RNAs processed by DICER which regulate gene expression at the post-transcriptional level, thereby modulating virtually all biological pathways^27–29^. Importantly, the RNAi pathway appears to be a particularly potent antiviral process, as many viruses have evolved several RNAi-suppressing strategies including encoding the viral suppressor of RNAi (VSR) proteins to minimize the RNAi pathway^30,31^. Therefore, compounds such as enoxacin which could serve as RNAi enhancers might be ideal candidates for antiviral therapy of COVID-19.

Studies have shown that enoxacin could enhance RNAi activity through its binding to, and stimulating, TAR RNA binding protein (TRBP), as the main cofactor of DICER, thereby facilitating the binding of DICER to target RNAs^32,33^. This enhancement in the RNAi pathway subsequently leads to a more potent RNAi (i.e. siRNA and miRNA) effect on the target RNAs, either mRNAs (targets of miRNAs) or viral RNAs (targets of the virally-derived siRNAs)^23,34,35^. Interestingly, enoxacin has recently been shown to exert a potent antiviral activity against several types of viruses such as Zika virus, Dengue virus, human immunodeficiency virus (HIV/AIDS), and langat virus in in vitro, organoid, and animal models by enhancing the RNAi pathway^36–39^, suggesting that the RNAi-enhancing activity of enoxacin could serve as a general antiviral strategy including against the novel coronavirus. Here, we exploited several in silico analyses to predict if enoxacin could exert anti-SARS-CoV-2 effects. Our results indicated that the RNA genome of SARS-CoV-2 might be processed by the endonuclealytic activity of DICER. Moreover, we determined a set of putative miRNA targets of enoxacin and found that a fraction of these miRNAs could restrict the entry of SARS-CoV-2 into the host cells by targeting key cell surface proteins. Another fraction of enoxacin-responsive miRNAs showed the potential to repress both host transcripts mediating the replication of the virus and viral transcripts encoding important viral proteins. Finally, other enoxacin-induced miRNAs appeared to potentially silence the cytokine storm driven by the SARS-CoV-2 infection as well as promote bronchiolar stem cell differentiation, thereby empowering the regeneration of the lung parenchyma. Overall, our findings strongly suggest that enoxacin and possibly other RNAi-enhancing members of the fluoroquinolones might serve as antiviral drugs able to be repositioned for effective COVID-19 therapy.

## Results

### The RNA genome of SARS-CoV-2 might be a suitable substrate for DICER

Since enoxacin has been reported to exert its anti-viral activity by means of enhancing the RNAi pathway through binding and stimulating the activity of TRBP, the physical partner of DICER^23,33^, we first investigated if the single-stranded RNA genome of SARS-CoV-2 might be processed by the RNAi machinery. As DICER acts on hairpin RNA structures, we used three methods to predict these precursor structures in the viral genome. The SM-based method, which predicts pre-miRNAs from given sequences using sequence-structure motif strategies^40^, predicted 145 hairpin structures and miRNA precursors potentially derived from the SARS-CoV-2 genome (Table S1), suggesting that DICER could act on the SARS-CoV-2 genome and gradually degrade it down to siRNAs. Moreover, we utilized two other hairpin-predicting tools, i.e. the Ab-initio and the BLASTN method (a miRBase feature), to examine if other different methods of hairpin prediction similarly identify potential DICER-acting genomic regions. Of note, in the Ab-initio method an approximation of miRNA hairpin structure is first searched for, before reconstituting the pre-miRNA structure^41^, while the BLASTN approach searches for a human miRNA homolog in the viral genome. Using these two approaches, we could predict 518 and 69 hairpin/miRNA precursors, respectively (Table S1), which could serve as binding sites for recruiting DICER and stimulating its RNase activity. Notably, comparing the results obtained with the three hairpin-predicting tools revealed that 12 hairpin structures were commonly predicted by all these approaches in nearly the same regions of the SARS-CoV-2 genome (Table 1). These results strongly suggest that the SARS-CoV-2 genome intrinsically harbors multiple hairpin structures which could be processed by DICER/TRBP complex, allowing for the efficient degradation of the coronavirus RNA genome through the RNAi pathway.

**Table 1:** Stem-loop structures co-predicted by three different methods in nearly the same genomic regions of the SARS-CoV-2.

	Ab-initio-based method		SM-based method		BLASTN method	
1	Sequences	Location	Sequences	Location	Sequences	Location
2	5′GCCUUUGGAGGCUGUGUGUUCUCUUAUGUUGGUUGCCAUAACAAGUGUGCCUAUUGGGUUCCACGUGCUAGCGCUAACAUAGGUUGUAACCAUACAGGUGUUGUUGGAGAAGGUUCCGAAGGU 3′	1498–1621	5′CUUUGGAGGCUGUGUGUUCUCUUAUGUUGGUUGCCAUAACAAGUGUGCCUAUUGGGUUCCACGUGCUAGCGCUAACAUAGGUUGUAACCAUACAGGUGUUGUU 3′	1479–1582	5′UCUUAUGUUGGUUGCCAUAACAAGUGUGCCUAUUGGGUUCCACGUGCUAGCGCUAA 3′	1499–1554
3	5′UGAACUUGAUGAAAGGAUUGAUAAAGUACUUAAUGAGAAGUGCUCUGCCUAUACAGUUGAACUCGGUACAGAAGUAAAUGAGUUCGCCUGUGUUGUGGCAGAUGCUGUCAUAAAAACUUUGCAACCAGUAUCUGAAUUA 3′	2832–2972	5′AAGUACUUAAUGAGAAGUGCUCUGCCUAUACAGUUGAACUCGGUACAGAAGUAAAUGAGUUCGCCUGUGUUGUGGCAGAUGCUGUCAUAAAAACUUUGCAA 3′	2816–2917	5′CACCACUGGGCAUUGAUUUAGAUGAGUGGAGUAUGG 3′	2884–2919
4	5′GUGAUACAUUCUGUGCUGGUAGUACAUUUAUUAGUGAUGAAGUUGCGAGAGACUUGUCACUACAGUUUAAAAGACCAAUAAAUCCUACUGACCAGUCUUCUUACAUCGUUGAUAGUGUUACAGUGAAGAAUGGUUCCAU 3′	7736–7876	5′CUGACCAGUCUUCUUACAUCGUUGAUAGUGUUACAGUGAAGAAUGGUUCCAUCCAUCUUUACUUUGAUAAAGCUGGUCAAAAGACUUAUGAAAGACAUUCUCU 3′	7715–7718	5′UUUGAUAAAGCUGGUCAAAAGACUUAUGAAAGACAUUCUCUCUCUCAUU 3′	7778–7826
5	5′AGUCUUCUUACAUCGUUGAUAGUGUUACAGUGAAGAAUGGUUCCAUCCAUCUUUACUUUGAUAAAGCUGGUCAAAAGACU 3′	7831–7911	5′UAAUAACACUAAAGGUUCAUUGCCUAUUAAUGUUAUAGUUUUUGAUGGUAAAUCAAAAUGUGAAGAAUCAUCUGCAAAAUCAGCGUCUGUUUACUACA 3′	7851–7949	5′AAAGGUUCAUUGCCUAUUAAUGUUAUAGUUUUUGAUGGUAAAUCAAA 3′	7862–7908
6	5′UAUUUUAGUGGAGCAAUGGAUACAACUAGCUACAGAGAAGCUGCUUGUUGUCAUCUCGCAAAGGCUCUCAAUGACUUCAGUA 3′	10051–10133	5′AACCACCACAAACCUCUAUCACCUCAGCUGUUUUGCAGAGUGGUUUUAGAAAAAUGGCAUUCCCAUCUGGUAAAGUUGAGGGUUGUAUGGUACAAGUAACUUG 3′	10016–10119	5′CUGGUAAAGUUGAGGGUUGUAUGGUACAAGUAACUUGUGGUACAACU 3′	10083–10129
7	5′CAGCUGAUGCACAAUCGUUUUUAAACGGGUUUGCGGUGUAAGUGCAGCCCGUCUUACACCGUGCGGCACAGGCACUAGUACUGAUGUCGUAUACAGGGCUUUUGACAUCUACAAUGAUAAAGUAGCUG 3′	13660–13710	5′AGGACGAAGAUGACAAUUUAAUUGAUUCUUACUUUGUAGUUAAGAGACACACUUUCUCUAACUACCAACAUGAAGAAACAAUUUAUAAUUUACUUAAGGAUUGU 3′	13615–13719	5′AGACACACUUUCUCUAACUACCAACAUGAAGAAACAAUUUAUAAUUUACUU 3′	13660–13710
8	5′CAGCUGAUGCACAAUCGUUUUUAAACGGGUUUGCGGUGUAAGUGCAGCCCGUCUUACACCGUGCGGCACAGGCACUAGUACUGAUGUCGUAUACAGGGCUUUUGACAUCUACAAUGAUAAAGUAGCUG 3′	13660–13710	5′AGGACGAAGAUGACAAUUUAAUUGAUUCUUACUUUGUAGUUAAGAGACACACUUUCUCUAACUACCAACAUGAAGAAACAAUUUAUAAUUUACUUAAGGAUUGU 3′	13615–13719	5′ACUUUCUCUAACUACCAACAUGAAGAA 3′	13366–13692
9	5′UGGCUUAUACCCAACACUCAAUAUCUCAGAUGAGUUUUCUAGCAAUGUUGCAAAUUAUCAAAAGGUUGGUAUGCAAAAGUAUUCUACACUCCAGGGACCACCUGGUACUGGUAAGAGUCA3′	17227–17348	5′GUAGUAGAAUUAUACCUGCACGUGCUCGUGUAGAGUGUUUUGAUAAAUUCAAAGUGAAUUCAACAUUAGAACAGUAUGUCUUUUGUACUGUAAA3′	17224–17318	5′GUUUUGAUAAAUUCAAAGUGAAUUCAACAUUAGAACAGUAUGUCUUUUGUACUGUA 3′	17261–17316
10	5′GAGGGUUUUUUCACUUACAUUUGUGGGUUUAUACAACAAAAGCUAGCUCUUGGAGGUUCCGUGGCUAUAAAGAUAACAGAACAUUCUUGGAAUGCUGAUCUUUAUAAGCUCAUGGGACACUUCGCAUGGUGGACAGCCUUU3′	21397–21539	5′CUUAAAUUAAGGGGUACUGCUGUUAUGUCUUUAAAAGAAGGUCAAAUCAAUGAUAUGAUUUUAUCUCUUCUUAGUAAAGGUAGACUUAUAAUUAGAGAAAA3′	21411–21512	5′GUGACUAUUGACUAUACAGAAAUUUCAUUUAUGCUUUGGUGUAAAGAUG 3′	21433–21471
11	5′AGAAUGUUCUCUAUGAGAACCAAAAAUUGAUUGCCAACCAAUUUAAUAGUGCUAUUGGCAAAAUUCAAGACUCACUUUCU3′	24646–24726	5′GUACUUGGACAAUCAAAAAGAGUUGAUUUUUGUGGAAAGGGCUAUCAUCUUAUGUCCUUCCCUCAGUCAGCACCUCAUGGUGUAGUCUUCUUG3′	24658–24751	5′CAAAAAGAGUUGAUUUUUGUGGAAAGGGCUAUCAUCUUAUGUCCUUC 3′	24672–24718
12	5′UACAUUUGGCUAGGUUUUAUAGCUGGCUUGAUUGCCAUAGUAAUGGUGACAAUUAUGCUUUGCUGUAUGACCAGUUGCUGUAGUUGUCUCAAGGGCUGUUGUUCUUGUGGAUCCUGCUGCAAAUUUG3′	25564–25691	5′CCUCAAAAAGAGAUGGCAACUAGCACUCUCCAAGGGUGUUCACUUUGUUUGCAACUUGCUGUUGUUGUUUGUAACAGUUUACUCACACCUUUUGCUCGUUGCUGCUGGCCUUGAAGCCC3′	25583–25702	5′UGUUGUUGUUUGUAACAGUUUACUCACACCUUUUGCUCGUUGCUGCU 3′	25643–25689

### Enoxacin might target the proteins mediating SARS-CoV-2 entry into host cells

**Figure 1:**
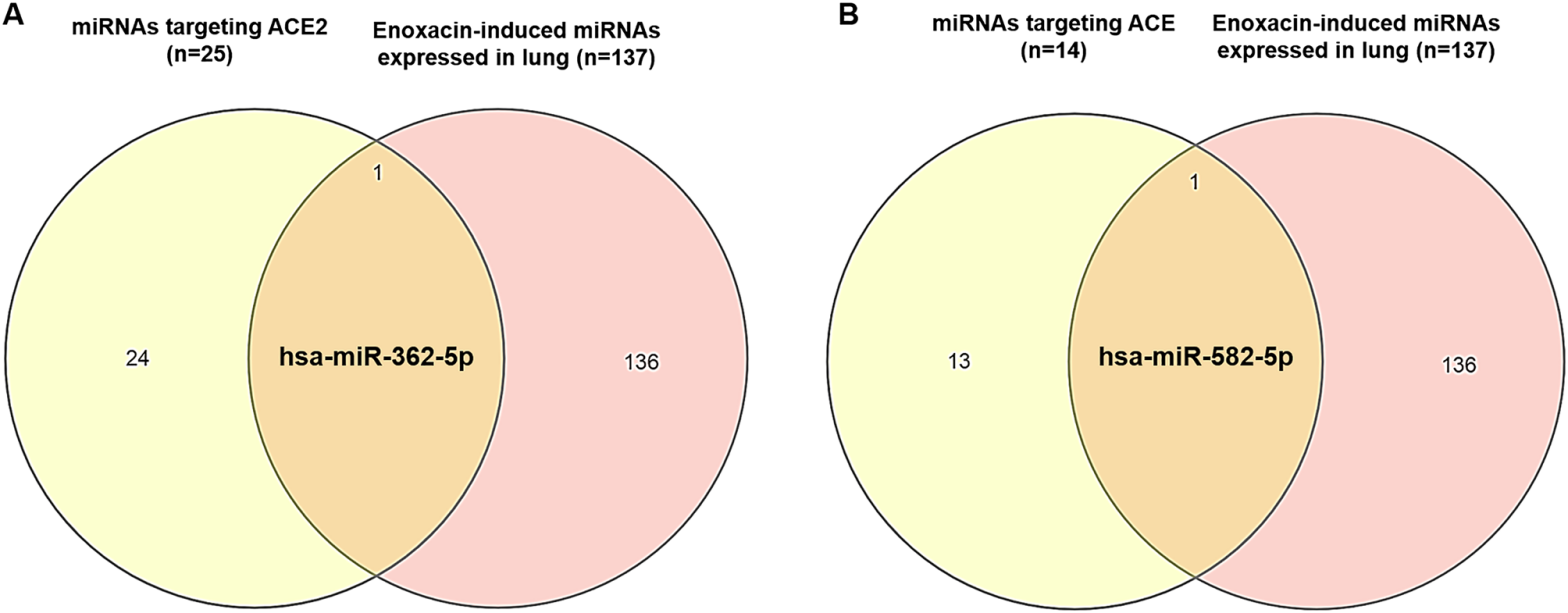
Venn diagram analysis of enoxacin-induced miRNAs and miRNAs targeting cell entry receptors necessary for SARS-CoV-2 infection. Entry receptors including ACE2 and ACE could be targeted by two enoxacin-induced miRNAs.

To predict if enoxacin-stimulated miRNAs could target SARS-CoV-2 entry receptors, we first sought to determine the miRNAs that are reported in several published research to be upregulated by enoxacin (Table S2). Analysis of enoxacin-induced miRNAs across multiple types of cell lines (i.e*.* prostate cancer cells, HEK cells, and melanoma cells) revealed that enoxacin could upregulate a large set of mature miRNAs. Indeed, 268 miRNAs were found to be upregulated under enoxacin treatment (Table S2). Comparing these 268 miRNAs with the miRNA expression profile of the lung tissue (see the list of miRNAs in Table S3) showed that 137 of enoxacin-upregulated miRNAs were also expressed in the lung tissue. To minimize the false positives, we used these 137 miRNAs (that were both enoxacin-induced and lung tissue-expressed) for our subsequent analyses. Next, we attempted to define miRNAs that could potentially target ACE2 as the main entry receptor of SARS-CoV-2 on cell surface as well as ACE as an alternative entry receptor of the virus (Table S4). To this end, we considered only those miRNAs which were co-predicted by three different miRNA target prediction tools (*i.e.* miRanda, TargetScan, and miRDB) in addition to the miRNAs on miRTarBase that have previously been experimentally verified to regulate these two cell-surface receptors. This stringent approach could considerably increase the reliability of our miRNA target analyses. Using this strategy, 25 and 14 miRNAs were found to potentially repress *ACE2* and *ACE* genes, respectively. Importantly, two of the miRNAs reported to be induced by enoxacin, i.e. hsa-miR-362-5p and hsa-miR-582-5p, were among these miRNAs that can target *ACE2* (Fig. 1A) and *ACE* (Fig. 1B), respectively, suggesting that enoxacin might be able to reduce the efficiency with which SARS-CoV-2 enters the host cells through cell surface receptors.

SARS-CoV-2 cell entry is reported to also depend on the activity of certain cell proteases particularly the transmembrane serine proteinase 2 (TMPRSS2) and 11D (TMPRSS11D), cathepsin L (CTSL), and FURIN. Judging from the analysis of miRNAs co-predicted or validated to target these proteases, we found they can be direct targets of a fraction of the enoxacin-induced miRNAs. Indeed, seven, two, three, and seven of the enoxacin-induced miRNAs were found to putatively target *TMPRSS2*, *CTSL*, *TMPRSS11D*, and *FURIN*, respectively (Table 2). These collective results highlight enoxacin's potential to restrict the entry of the novel coronavirus into the cells.

**Table 2:** Enoxacin-induced miRNAs that can target membranous proteases facilitating SARS-CoV-2 entry.

Target transcripts	Targeting miRNAs
*TMPRSS2*	hsa-miR-582-5p, hsa-miR-452-5p, hsa-miR-214-3p, hsa-miR-1208, hsa-miR-181b-5p, hsa-miR-181c-5p, hsa-miR-98-5p
*TMPRSS11D*	hsa-miR-574-3p, hsa-miR-186-5p, hsa-miR-23a-3p
*CTSL*	hsa-miR-501-5p, hsa-miR-518a-5p
*FURIN*	hsa-miR-20a-5p, hsa-miR-483-3p, hsa-miR-17-5p, hsa-miR-4286, hsa-miR-140-3p, hsa-miR-497-5p, hsa-miR-107

### Enoxacin might target intracellular proteins interacting with SARS-CoV-2 components

**Figure 2:**
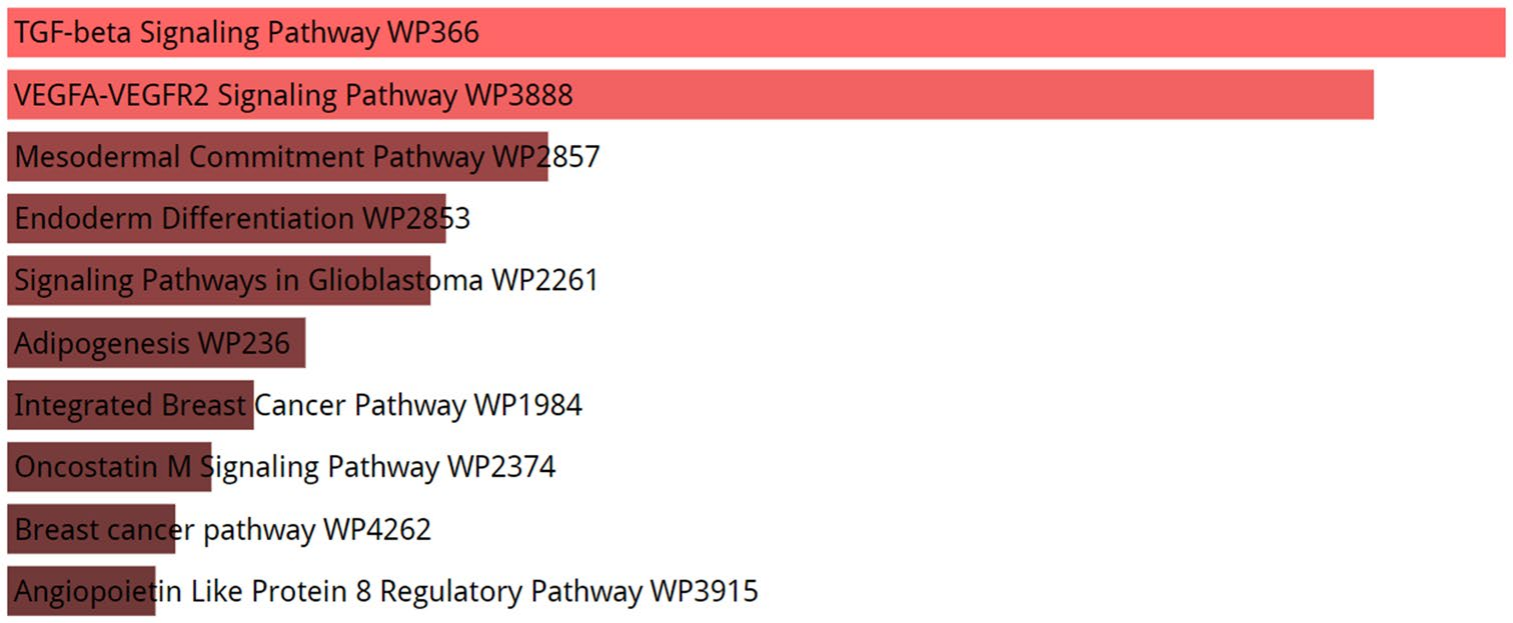
Enrichment analysis of genes which are predicted to be targeted by enoxacin-induced miRNAs. The Panther enrichment analysis using Enrichr showed that genes putatively targeted by enoxacin-induced miRNAs were mostly involved in TGF-β signaling (p-value = 5.782e−20). The lighter the red color is, the more significant the p-value.

**Figure 3:**
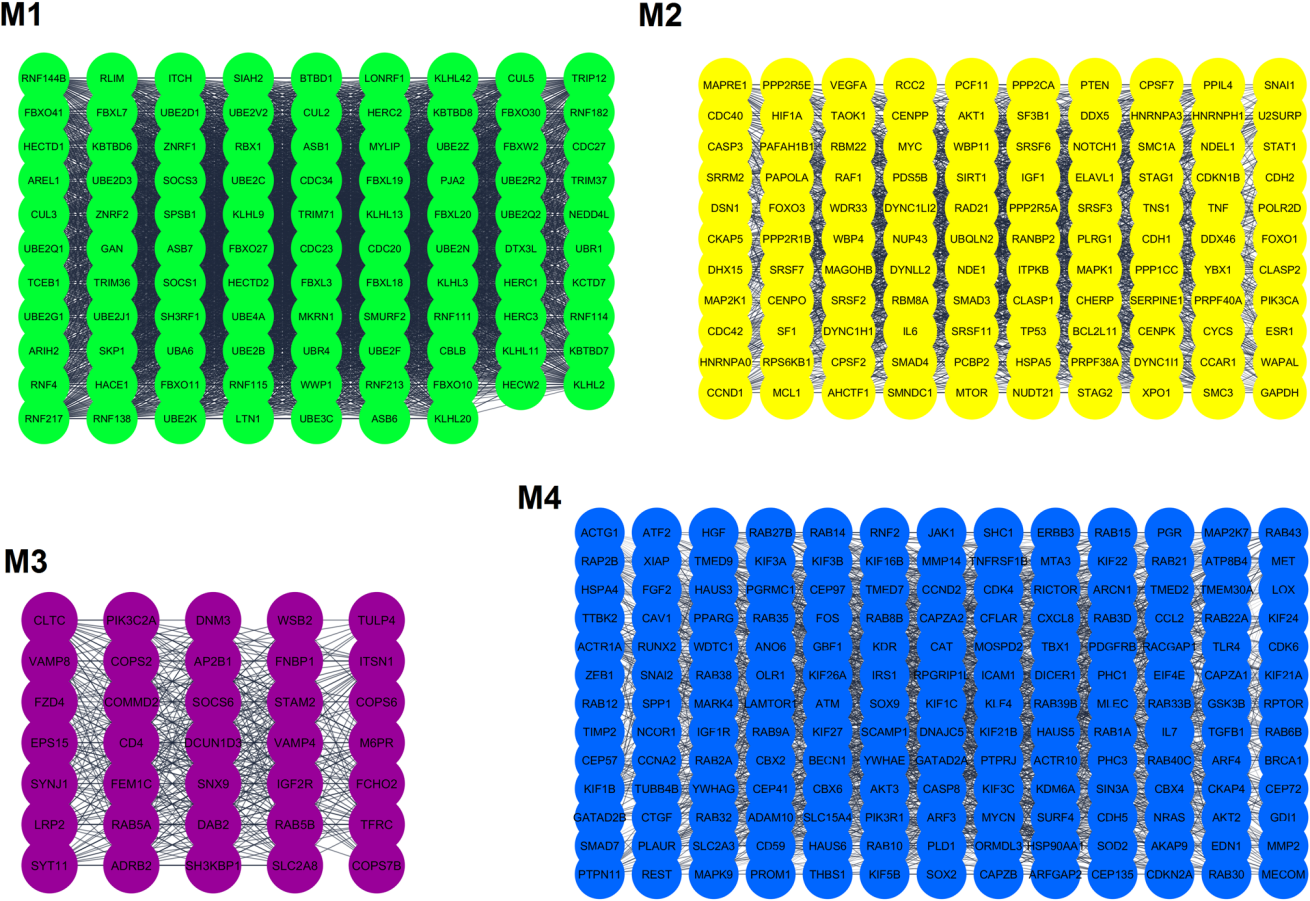
The PPI network (top four modules) of genes potentially targeted by enoxacin-induced miRNAs. The PPI network of genes predicted to be targeted by enoxacin-induced miRNAs were depicted by Cytoscape (only interactions with the confidence of a combined score > 0.400 were included) and protein modules were identified by MCODE (cutoff criteria were ‘degree cutoff = 2’, ‘k-core = 2’, ‘node score cutoff = 0.2’, and ‘maximum depth = 100). M: Module.

**Figure 4:**
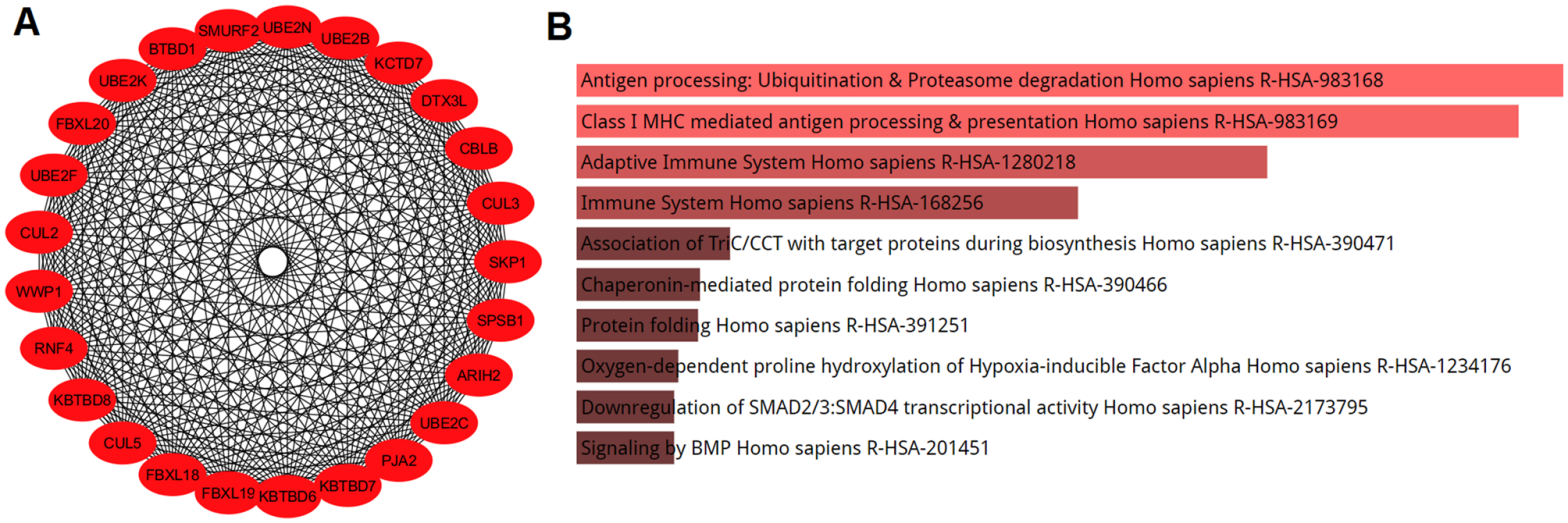
Hub genes in the PPI network could be targeted by enoxacin-induced miRNAs. (**A**) Twenty-five hub genes were identified by Cytohubba and MCC method. (**B**) The KEGG pathway enrichment analysis showed that these genes were mostly associated with MHC class I-mediated antigen processing (p-value = 1.698e−86). The lighter the red color is, the more significant the p-value.

To perform enrichment analysis on the SARS-CoV-2-interacting host genes that might be affected by enoxacin, we used the union of co-predicted (from miRanda, PITA, TargetScan, and miRDB) and validated targets (from miRTarBase) of the miRNAs upregulated by enoxacin. Next, to find out if these targets are also expressed in the lung tissue (to enhance the reliability of our in silico analysis), the intersection of these target genes and the genes expressed in the lung tissue was determined (see the list of mRNA transcripts in Table S3). Then, the PPI network of these targets was extracted using STRING database. Totally, 3893 genes were predicted to be targeted by enoxacin-upregulated miRNAs in the lung (Table S5). As shown in Fig. 2, the pathway enrichment analysis of these genes revealed that the TGF-β signaling pathway was the most significant pathway targeted by enoxacin (p-value = 5.782e−20). In addition, we determined the top four protein modules (M1 to M4) in the PPI network of the targeted genes (Fig. 3). The pathway enrichment analysis of these modules (Table 3) highlighted that M1 was related to MHC class I-mediated antigen processing and TGF-β signaling pathway; M2 was mainly associated with M phase pathway and PI3K-Akt signaling; M3 was primarily consisted of endocytosis and EGF/EGFR signaling; and the M4 module highlighted the involvement of VEGF/VEGFR2 and PI3K-Akt pathways (Table 3). Furthermore, as shown in Fig. 4, the enrichment analysis of the top 25 hub genes identified by Cytohubba showed that hub genes mostly belonged to MHC class I-mediated antigen presentation (p-value = 7.684e−25), suggesting that enoxacin might modulate the immune system. It is also noteworthy that we depicted the PPI network for 386 genes which were observed to be simultaneously co-targeted by 10 or more enoxacin-induced miRNAs and repeated all the above analyses. Our data revealed a highly similar set of results (data not shown), suggesting that the overall impact of enoxacin might be mostly mediated by a fraction of its target miRNAs.

**Table 3:** GO and pathway enrichment analysis of top four modules in the PPI network of genes predicted to be targeted by enoxacin-induced miRNAs.

Modules	Classification system	Pathways	p-value
1	Bioplanet 2019	Antigen presentation: folding, assembly, and peptide loading of class I MHC proteins	1.698e−86
	WikiPathways 2019	TGF-β signaling pathway	3.646e−6
	KEGG	Ubiquitin-mediated proteolysis	1.510e−60
2	Bioplanet 2019	M phase pathway	1.226e−39
	WikiPathways 2019	PI3K-Akt signaling pathway	1.170e−17
	KEGG	Splicesome	2.026e−23
3	Bioplanet 2019	Endocytosis	2.710e−14
	WikiPathways 2019	EGF/EGFR signaling pathway	3.439e−7
	KEGG	Endocytosis	2.291e−13
4	Bioplanet 2019	Pathways in cancer	4.469e−17
	WikiPathways 2019	VEGF/VEGFR2 signaling pathway	4.704e−18
	KEGG	PI3K-Akt signaling pathway	2.333e−19

On the other hand, we used the BioGRID database to obtain human proteins that interact with SARS-CoV-2 proteins (Table S6) for which the PPI network was depicted (Fig. S1A). The results of KEGG pathway enrichment analysis (Fig. S2A) revealed that these 321 proteins were involved in protein processing in endoplasmic reticulum and RNA transport (p-value = 0.0001441), which highlights potential intracellular machineries facilitating SARS-CoV-2 infection. In addition, the top four modules of the interaction network of these human proteins (Fig. S1B) were observed to be mostly involved in RNA transport and AMPK signaling pathway (enrichment analysis by KEGG, p-value = 2.651e−5 and 1.956e−4 respectively). (Fig. S2B). Furthermore, analysis of the nine hub proteins from host cells interacting with SARS-CoV-2 proteins (Fig. S3) demonstrated that they were associated with insulin signaling and cell junction (enrichment analysis by KEGG, p-value = 0.001625 and 0.002486, respectively).

The proteins and cellular pathways described above appear to interact with SARS-CoV-2 viruses, thereby facilitating its infection. We, therefore, hypothesized that enoxacin might down-regulate some of these host proteins to perturb the viral life cycle. Our analysis revealed that enoxacin-upregulated miRNAs could target 103 of these 321 proteins (Table S7). Taken together, the induction of certain miRNAs by enoxacin could lead to the repression of host proteins mediating the infection of the novel coronavirus.

### A set of miRNAs targeting SARS-CoV-2 components are upregulated by enoxacin

A fraction of enoxacin-upregulated miRNAs might directly inhibit the viral RNA genome. The miRDB search tool predicted 900 miRNAs that might target the SARS-CoV-2 RNA genome (Table S8). Venn diagram analysis showed that 26 out of these miRNAs (with target score > 70) can be upregulated by enoxacin. These 26 miRNAs were predicted to directly bind and target various regions of the SARS-CoV-2 RNA genome which encode key viral components (Table S9); notably, a fraction of these miRNAs appear to target multiple viral components simultaneously (Table 4). Furthermore, we compared the enoxacin-induced miRNAs with the miRNAs previously reported to target SARS-CoV-2 genome via different prediction methods^42–44^. Venn diagram analysis revealed that hsa-miR-455-5p, hsa-miR-623, hsa-miR-193a-5p, hsa-miR-602, hsa-miR-222, hsa-miR-378a-3p, hsa-miR-34a-5p, and hsa-miR-98-5p which were reported in these studies to target SARS-CoV-2 genome, were among miRNAs upregulated by enoxacin (Fig. S4). We, therefore, conclude that the RNA genome of the SARS-CoV-2 might be regulated by certain human miRNAs that are enoxacin-inducible.

**Table 4:** SARS-CoV-2 components can be targeted by enoxacin-induced miRNAs.

SARS-CoV-2 components	Targeting miRNAs
NSP1	hsa-miR-125b-2-3p, hsa-miR-382-5p
NSP2	hsa-miR-513a-3p, hsa-miR-376a-3p, hsa-miR-583, hsa-miR-186-5p, hsa-miR-495-3p, hsa-miR-3065-5p, hsa-miR-125-2-3p
NSP3	hsa-miR-485-3p, hsa-miR-20a-3p, hsa-miR-23a-3p, hsa-miR-520a-3p, hsa-miR-376a-3p, hsa-miR-452-5p, hsa-miR-382-5p, hsa-miR-576-5p, hsa-miR-583, hsa-miR-181c-5p, hsa-miR-181b-5p, hsa-miR-497-5p, hsa-miR-186-5p, hsa-miR-545-3p, hsa-miR-30b-5p, hsa-miR-505-3p, hsa-miR-518a-5p, hsa-miR-29c-3p, hsa-miR-29b-3p, hsa-miR-495-3p, hsa-miR-29a-3p, hsa-miR-194-5p, hsa-miR-3065-5p, hsa-miR-125b-2-3p
NSP4	hsa-miR-3065-5p, hsa-miR-125b-2-3p, hsa-miR-107, hsa-miR-513a-3p, hsa-miR-382-5p, hsa-miR-583, hsa-miR-181c-5p, hsa-miR-181b-5p, hsa-miR-186-5p, hsa-miR-30b-5p, hsa-miR-518a-5p, hsa-miR-29a-3p, hsa-miR-29b-3p, hsa-miR-495-3p, hsa-miR-29a-3p, hsa-miR-194-5p
NSP6	hsa-miR-485-3p, hsa-miR-583, hsa-miR-181c-5p, hsa-miR-181b-5p, hsa-miR-30b-5p, hsa-miR-518a-5p, hsa-miR-29c-3p, hsa-miR-29b-3p, hsa-miR-495-3p, hsa-miR-29a-3p, hsa-miR-194-5p
NSP7	hsa-miR-518a-5p
NSP8	hsa-miR-20a-3p, hsa-miR-382-5p, hsa-miR-576-5p, hsa-miR-181c-5p, hsa-miR-181b-5p
NSP9	hsa-miR-495-3p, hsa-miR-194-5p
ORF3a	hsa-miR-3065-5p, hsa-miR-497-5p, hsa-miR-545-3p, hsa-miR-518a-5p
ORF5	hsa-miR-107
ORF6	hsa-miR-513a-3p
ORF7a	hsa-miR-452-5p, hsa-miR-186-5p, hsa-miR-125b-2-3p
ORF8	hsa-miR-181c-5p, hsa-miR-181b-5p, hsa-miR-30b-5p, hsa-miR-513a-3p, hsa-miR-376a-3p
Nucleocapsid	hsa-miR-497-5p, hsa-miR-545-3p, hsa-miR-29c-3p, hsa-miR-29b-3p, hsa-miR-29a-3p, hsa-miR-107, hsa-miR-513a-3p, hsa-miR-20a-3p, hsa-miR-382-5p
RdRp	hsa-miR-186-5p, hsa-miR-545-3p, hsa-miR-505-3p, hsa-miR-495-3p, hsa-miR-3065-5p, hsa-miR-125b-2-3p, hsa-miR-107, hsa-miR-23a-3p, hsa-miR-520a-3p, hsa-miR-376a-3p hsa-miR-452-5p, hsa-miR-382-5p, hsa-miR-497-5p
Spike	hsa-miR-518a-5p, hsa-miR-29c-3p, hsa-miR-29b-3p, hsa-miR-495-3p, hsa-miR-29a-3p, hsa-miR-3065-5p, hsa-miR-125b-2-3p, hsa-miR-107, hsa-miR-513a-3p, hsa-miR-485-3p, hsa-miR-20a-3p, hsa-miR-23a-3p, hsa-miR-376a-3p, hsa-miR-382-5p, hsa-miR-576-5p, hsa-miR-497-5p, hsa-miR-186-5p, hsa-miR-545-3p
Helicase	hsa-miR-29c-3p, hsa-miR-29b-3p, hsa-miR-29a-3p, hsa-miR-194-5p, hsa-miR-3065-5p, hsa-miR-513a-3p, hsa-miR-452-5p, hsa-miR-382-5p, hsa-miR-576-5p, hsa-miR-30b-5p, hsa-miR-505-3p, hsa-miR-518a-5p
2OMT	hsa-miR-29c-3p, hsa-miR-29b-3p, hsa-miR-495-3p, hsa-miR-29a-3p, hsa-miR-194-5p, hsa-miR-513a-3p, hsa-miR-20a-3p, hsa-miR-576-5p, hsa-miR-181c-5p, hsa-miR-181b-5p, hsa-miR-186-5p
3′-5′ exonuclease	hsa-miR-29c-3p, hsa-miR-29b-3p, hsa-miR-495-3p, hsa-miR-29a-3p, hsa-miR-376a-3p, hsa-miR-181c-5p, hsa-miR-181b-5p, hsa-miR-30b-5p, hsa-miR-505-3p
3C-like proteinase	hsa-miR-495-3p, hsa-miR-194-5p, hsa-miR-3065-5p, hsa-miR-125b-2-3p, hsa-miR-485-3p, hsa-miR-376a-3p, hsa-miR-576-5p, hsa-miR-583, hsa-miR-30b-5p, hsa-miR-505-3p
endoRNAse	hsa-miR-513a-3p, hsa-miR-376a-3p, hsa-miR-497-5p, hsa-miR-30b-5p, hsa-miR-495-3p, hsa-miR-3065-5p, hsa-miR-513a-3p
5′-UTR	hsa-miR-505-3p

### Enoxacin-enhanced miRNAs may exert immunomodulatory effects against the SARS-CoV-2-induced cytokine storm

An exaggerated immune response in the respiratory system to the SARS-CoV-2 infection has been suggested to contribute to the high mortality rate seen in patients with COVID-19^9,45^. To predict if enoxacin could attenuate such cytokine storms in patients with COVID-19, we extracted the anti-inflammatory miRNAs through literature review and found 25 miRNAs frequently reported to exert immunomodulatory effects^46,47^. Interestingly, our data showed that nine out of these 25 miRNAs including hsa-miR-21, hsa-miR-17, hsa-miR-146a, hsa-miR-155, hsa-miR-181b, hsa-miR-181c, hsa-miR-31, hsa-miR-92a, and hsa-miR-223 could be upregulated by enoxacin (Fig. S5). Of note, we noticed that some of the enoxacin-upregulated miRNAs targeted PIK3CA and GSK3B as major components of PI3K signaling which plays a key role in driving inflammatory responses (data not shown). In conclusion, enoxacin might prove beneficial in fighting the SARS-CoV-2 infection via promoting immunomodulatory effects.

### Enoxacin might promote bronchiolar stem cell differentiation, reversing viral negative effects on lung parenchyma

As with SARS-CoV infection (which can promote the severe acute respiratory syndrome, SARS), the SARS-CoV-2 infection triggers and promotes lung injury as a typical symptom of hospitalized COVID-19 patients^48^. Cumulative evidence suggests that the bronchio-alveolar stem cells (BASCs), which are characterized by “Sca-1^+^ CD34^+^ CD45^−^ Pecam^−^” markers, participate in tissue regeneration after lung injury^49^. Importantly, these cells appear to be a prime target of SARS-CoV infection^50^. To predict if miRNAs upregulated by enoxacin might play a role in lung regeneration, we extracted 95 previously reported miRNAs targeting the developmental stage-specific transcription factors and key marker genes of BASCs^50^. Venn diagram analysis showed that 27 out of these miRNAs could be upregulated by enoxacin treatment (Table S10). Particularly, hsa-let-7d, hsa-let-7g, and hsa-let-7c (predicted to target CD34^50^) were among the miRNAs upregulated by enoxacin. Thus, enoxacin might induce BASC differentiation (necessary for lung tissue repair upon injury) by upregulating certain miRNAs.

## Discussion

The RNAi enhancer enoxacin has been proposed as a repurposed drug candidate for targeting multiple cancers^51,52^ and several viral diseases^36,37,53,54^. For example, Yan-Peng et al. reported that enoxacin augmented virus-derived siRNA levels in Zika virus-infected human neural progenitor cells and brain organoids, highlighting that enoxacin can promote viral RNA-genome degradation^36^. We hypothesized that the RNAi-enhancing activity of enoxacin might similarly interfere with SARS-CoV-2 infection. In line with this, Bartoszewski et al. suggested that SARS-CoV-2 may act through the depletion of specific host miRNAs^55^, explaining, at least partly, why enhancing the host-cell miRNA activity might be a viable therapeutic option against SARS-CoV-2 replication. In addition, Chow et al. found that most of the differentially expressed miRNAs in Calu3 cells infected with SARS-CoV-2 were downregulated^56^. Interestingly, we found that some of these down-regulated miRNAs such as hsa-miR-194-5p, hsa-miR-21-5p, and hsa-miR-940 can be upregulated by enoxacin (see Table S2).

Moreover, we found hundreds of stem-loop structures in the RNA genome of SARS-CoV-2 which could be binding sites for the DICER/TRBP complex as the direct target of enoxacin's stimulatory effect. Several small RNAs have experimentally been found to be derived from our predicted stem-loop regions of the SARS-CoV-2 genome. Merino et al. reported eight SARS-CoV-2-derived small RNA molecules experimentally confirmed by small-RNA sequencing in the SARS-CoV-2-infected human Calu-3 cells^57^. Interestingly, six of these genuine SARS-CoV-2 small RNAs were predicted using the SM-based or Ab-initio methods (see Table S1) to be derived from the SARS-CoV-2 genomic regions which included the regions 396–496, 555–634, 26742–26873,27002–27104 (predicted by the SM-based approach), 26903–27016, and 29541–29625 (predicted by the Ab-initio approach). The identification of these putative DICER binding sites in the SARS-CoV-2 genome is important for the targeting of viral RNA genome, as intramolecular stem-loop structures in RNA molecules are known to be typical substrates for Dicer enzymes^58,59^. Finally, the fact that the SARS-CoV-2 genome encodes VSRs (i.e. nucleocapsid and SARS-CoV-2-7a proteins) further supports the importance of the RNAi pathway as a crucial antiviral defense mechanism in mammalian cells^60,61^.

In addition to the direct action of DICER/TRBP complex on the SARS-CoV-2 genome, our results suggested that 26 enoxacin-induced miRNAs could target different regions of the SARS-CoV-2 genome (see Table S9). Experimentally, eight out of these 26 miRNAs have previously been reported to target SARS-CoV-2 genome^42–44^, providing further support for our in silico findings. Altogether, these findings suggest the RNAi pathway as an effective antiviral mechanism against various viruses including coronaviruses, and support the application of the RNAi enhancer enoxacin in potential inhibition of the SARS-CoV-2 infection.

Targeting viral entry receptors and cell-membrane-associated proteases necessary for SARS-CoV-2 infection has been proposed as a rational strategy to treat COVID-19^62^. Our results showed that some of enoxacin-induced miRNAs could potentially target the cell-surface receptors and cell-membrane-associated proteases necessary for SARS-CoV-2 infection. In this regard, hsa-miR-214 and has-miR-98, which we found to potentially target *TMPRSS2* using several miRNA target prediction and validation databases, have been experimentally verified to target *TMPRSS2* in Caco-2, HMVEC-L, and HUVEC cells^63,64^. Finally, microarray analyses^65^ support our data regarding the high likelihood of hsa-miR-107 to repress *FURIN*, which codes for another cell-membrane-associated protease involved in SARS-CoV-2 pathogenesis^66^.

Following the entry of SARS-CoV-2 into the lung epithelial cells, these cells start secreting inflammatory factors to recruit various leukocytes to the lung tissue, helping to suppress the infection^67,68^. However, the over-production of inflammatory mediators might lead to the so-called acute respiratory distress syndrome (ARDS) which promotes the destruction of the lung parenchyma in patients with severe COVID-19^69^. Among these inflammatory mediators, IL-6 is strikingly upregulated in the blood samples from non-survivor individuals^69^, explaining why the IL-6 inhibitor Tocilizumab is currently being used in patients with ARDS^9^.

Notably, we found that nine of the enoxacin-induced miRNAs had previously been found to exert immunomodulatory effects^46^. The probable immunomodulatory effect of enoxacin is not surprising given that several reports have independently shown the immunomodulatory activities of fluoroquinolones in diverse contexts^22^. It is worthwhile to note that the enoxacin-enhanced miRNAs hsa-miR-21, hsa-miR-146a, hsa-miR-92a, hsa-miR-181b, and hsa-miR-223 have been reported to induce anti-inflammatory effects in part by decreasing IL-6 expression^70–74^. Moreover, overexpression of hsa-miR-21 in lipopolysaccharide-induced macrophages was reported to significantly decrease IL-6 and increase anti-inflammatory IL-10 secretion^70^. Further, hsa-miR-146a overexpression in human retinal endothelial cells or in lipopolysaccharide-induced macrophages reduced the IL-6 secretion^75,76^. Furthermore, hsa-miR-146s can negatively regulate TNFα-induced inflammatory pathway in macrophages^77^ and reduce IL-6 secretion in primary human small airway epithelial cells^78^. Another enoxacin-induced miRNA, hsa-miR-92a, was found to directly target mitogen-activated protein kinase kinase 4, decreasing TNFα and IL-6 production in macrophages^79^. Interestingly, hsa-miR-181b was found to decrease IL-6 expression in lipopolysaccharide-induced macrophages^80^. Finally, hsa-miR-223, a crucial regulator of the innate immune responses, was found to directly target poly (adenosine diphosphatase-ribose) polymerase-1 (PARP-1)^81^ and NLRP^82^, thereby suppressing inflammation. Taken together, these collective evidence strongly suggest that the miRNAs induced by enoxacin are functionally involved in dampening inflammation in various biological contexts, and that the potential enoxacin-driven immunomodulation might play an important part in the effective treatment of COVID-19 patients.

We also found that a fraction of the enoxacin-induced miRNAs particularly hsa-let-7c, hsa-let-7g, and hsa-let-7d could contribute to BASC differentiation possibly by targeting the stem cell marker CD34^50^. In accordance with this result, over-expression of hsa-let-7 family members has frequently been reported to inhibit various stem cell states and promote multi-lineage differentiation^27,83,84^. BASCs were found to be activated upon different lung injuries, and differentiate into multiple cell lineages for lung regeneration^85^. Since alveolar damage and pulmonary fibrosis are the main pathological findings in patients with severe COVID-19^86^, triggering lung stem cell pools to differentiate may enhance lung repair and regeneration^85^. In addition, Mallick et al. reported that 15 miRNAs were downregulated in SARS-CoV-infected BASCs^50^, of which the expression of hsa-let-7c, hsa-let-7d, hsa-let-7g, hsa-miR-186, hsa-miR-98, and hsa-miR-223 can be restored by enoxacin-induced miRNAs (see Table S2).

The PPI network the target genes of enoxacin-induced, lung-expressed miRNAs revealed that these genes were mainly associated with MHC class I-mediated antigen processing, TGF-β signaling pathway, PI3K-AKT signaling, and endocytosis. In this regard, Xia et al.^87^ demonstrated in mice that MHC class I exacerbated the vesicular stomatitis virus (VSV)-induced infection in the lung by disrupting the type I IFN signaling, and that the viral load in MHC class I-deficient macrophages was decreased. Moreover, the possible inhibitory effect of enoxacin on TGF-β signaling is interesting because TGF-β signaling (i) drives the chronic adaptive immune responses in patients with ARDS^88^, resulting in rapid and massive edema and fibrosis in these patients (for this reason, its blockade has been suggested as a potential treatment of COVID-19)^89^ and (ii) increases *FURIN* expression in well-differentiated primary human bronchial epithelial cells^90^ (therefore, its blockade may attenuate the SARS-CoV-2 entry into the cells). In addition, the inhibitors of PI3K-AKT signaling, which we suggest to be targeted by enoxacin, have been proposed as drug candidates in COVID-19 treatments^91^. This argument is supported by the point that PI3K-AKT signaling is required for establishing persistent SARS-CoV infection in Vero E6 cells^92^. The possible effect of enoxacin on endocytosis pathway is also notable as some proteins of this pathway are interactors of SARS-CoV-2 proteins^93^. Overall, it seems that enoxacin upregulates a specific set of miRNAs in lung cells which can restrict the entry, replication, and infection of SARS-CoV-2 through suppressing several intracellular pathways.

It is also noteworthy that our study has a number of limitations. First, enoxacin-induced miRNA profiles were extracted from studies on cancer cells which may not completely reflect the actual effects of enoxacin on SARS-CoV-2-infected cells, although the molecular interaction of enoxacin with its target, TRBP, does not appear to be mechanistically different in different types of cell. To minimize this limitation, we considered only those enoxacin-induced miRNAs that were also expressed by lung cells. Second, our in silico results need to be investigated experimentally in both in vitro and animal models of SARS-CoV-2 infection, although we provided a large body of experimental evidence from previously published research for our in silico findings. Altogether, our analysis strongly suggests that enoxacin could be a promising drug candidate for COVID-19 treatment.

## Conclusion

**Figure 5:**
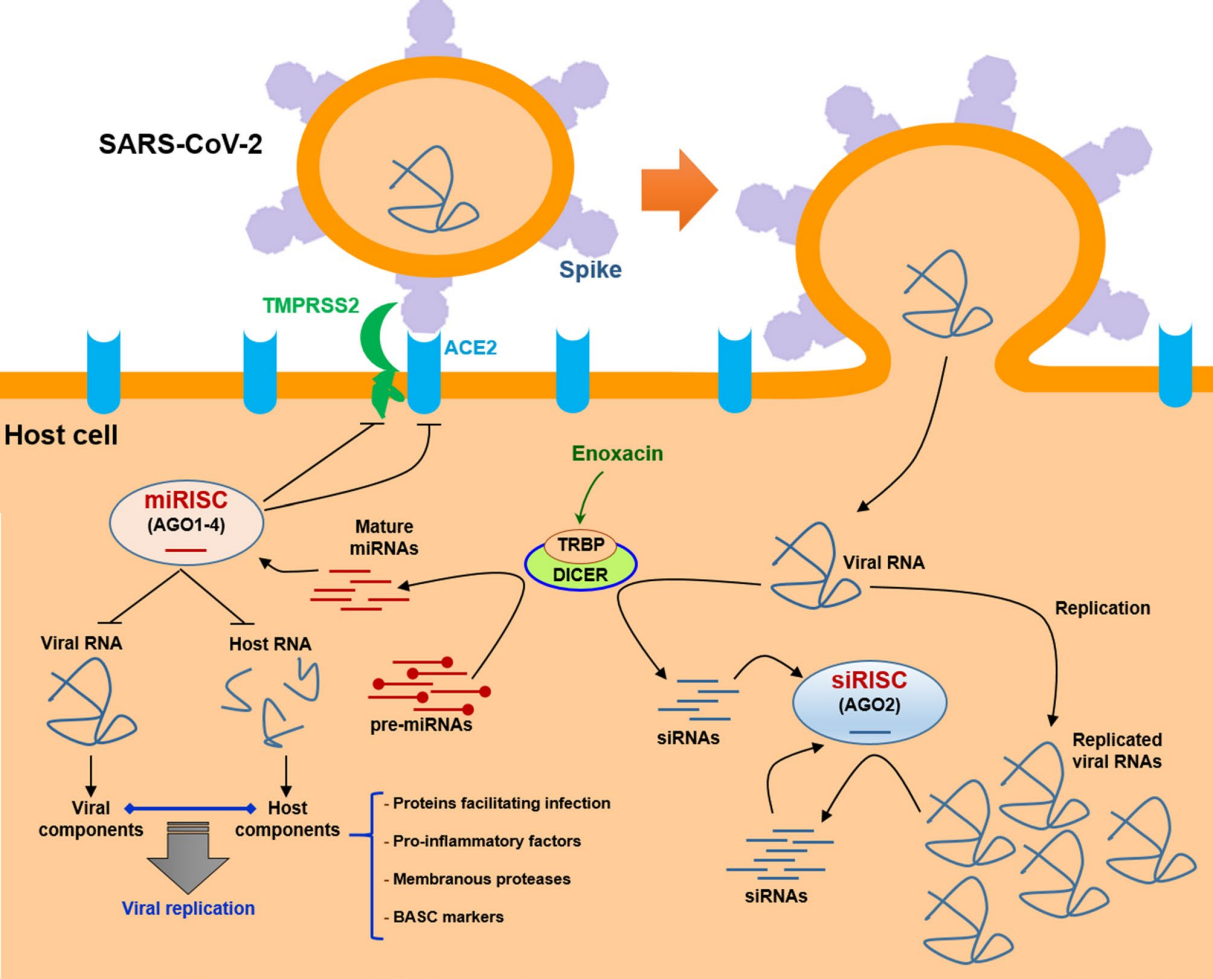
Modeling of the potential SARS-CoV-2 inhibition by enoxacin. Enoxacin enhances the RNAi pathway through binding to TRBP, the physical partner of DICER. This interaction enhances the dicing of viral RNA genome directly by DICER as well as upregulates certain mature miRNAs which could target SARS-CoV-2 RNA genome and viral transcripts including VSRs through RISC complexes. Enoxacin-induced miRNAs might also target entry receptors and membranous proteases in host cells, pro-inflammatory genes in the immune cells, and stem cell markers in BASCs. It might also suppress the interactions between certain viral and host RNA molecules which mediate and facilitate viral replication and infection. ( ) designates mutual interaction. miRISC: miRNA-induced silencing complex; siRISC: siRNA-induced silencing complex.

Enoxacin belongs to the fluoroquinolone family of synthetic antibiotics which was recently found to enhance the maturation of TRBP/DICER-dependent small RNAs, leading to a global increase in the concentration of these regulatory RNAs. The enhancement of the RNAi pathway by enoxacin has been frequently found to exert detrimental effects on the replication of several types of viruses, suggesting that enoxacin might similarly inhibit the infection caused by the novel coronavirus. Using several in silico analyses, we observed that enoxacin could promote the DICER/TRBP-mediated degradation of the SARS-CoV-2 RNA genome, as our data suggested that the viral genomic RNA can be a suitable substrate for the DICER activity. We could also find several enoxacin-upregulated miRNAs that are predicted to directly target the viral genome. Importantly, several enoxacin-induced miRNAs were suggested to inhibit not only the viral components mediating viral entry into host cells and its intracellular replication but could also target certain host proteins interacting with the viral components. Therefore, enoxacin might be able to exert antiviral effects against the SARS-CoV-2 infection. Figure 5 illustrates a schematic model of how the SARS-CoV-2 infection might be suppressed by the application of enoxacin. The potential antiviral effects of enoxacin could be probably further increased when enoxacin treatment is accompanied by the delivery of a shRNA sequence directly targeting the SARS-CoV-2 genome. In this way, not only enoxacin could restrict the viral replication per se as suggested in this study but also can enhance the processing of the delivered shRNA in order to provide a more potent inhibitory effect on the novel coronavirus. Finally, since there are other fluoroquinolones which similarly exhibit RNAi enhancing effects, it might be possible to also use those fluoroquinolone members for targeting the novel coronavirus infection. Further investigations are needed to examine how enoxacin or other family members might modulate the infection caused by SARS-CoV-2.

## Materials and Methods

### Prediction of miRNA- and shRNA/siRNA precursors in the SARS-CoV-2 genome

The shRNA/miRNA precursor structures in the SARS-CoV-2 genome were predicted by three web server tools. First, the ab initio method was used to predict miRNA hairpin structures in the entire 29,903-nucleotide genome of SARS-CoV-2 (NC_045512.2) using miRNAFold with default parameters^41,94^. The entire SARS-CoV-2 genome was also analyzed by the sequence-structure motif-based (SM-based) miRNA prediction method using the Regulatory RNA web tool with default parameters (http://www.regulatoryrna.org/webserver/SSMB/pre-miRNA/home.html). The BLASTN method was also run to predict precursor structures using the miRBase search tool^95^.

### Obtaining the list of enoxacin-induced miRNAs and prediction of their target genes

The lists of enoxacin-induced miRNAs were obtained from previous experiments analyzing the effects of enoxacin on human embryonic kidney cells (HEK293)^33^, human prostate cancer cell lines (LNCaP and DU145)^34^, and the human melanoma cell line A375^35^. All miRNAs induced by enoxacin across different cell lines were extracted using Venn diagram. The expression profiles of miRNAs and mRNAs of the human lung tissue were obtained from the IMOTA database, an interactive multi-omics-tissue atlas^96^. The target genes of miRNAs were co-predicted by four databases (miRDB^97^, PITA^98^, miRanda^99^, and TargetScan^100^) provided by miRWalk^101^. To further increase the likelihood of obtaining true-positive miRNA targets, only transcripts with at least two binding sites for any given miRNA were extracted from the miRWalk 2.0 atlas^101^. Validated target genes (FDR < 0.05) of enoxacin-induced miRNAs were obtained from the miRTarBase database^102^ via the MIENTURNET web tool^103^.

### Gene ontology (GO) and biological pathway analyses

Gene set enrichment analysis of the predicted target genes for up-regulated miRNAs was performed using Enrichr as an enrichment analysis web application which provides access to 35 gene-set libraries^104^. Several features of the Enrichr database including KEGG, Wikipathways, and BioPlanet were used for GO analysis. P < 0.05 was considered to indicate statistical significance and the results were ranked by P-value.

### Protein–protein interaction (PPI) network analysis, module selection, and identification of hub genes

The PPI networks were constructed to infer interaction among proteins using the online STRING database (http://string-db.org/). Interactions with the confidence of a combined score > 0.400 were imported into Cytoscape to construct the PPI network. We used MCODE to identify the modules in the PPI network^105^. The cutoff criteria were ‘degree cutoff = 2’, ‘k-core = 2’, ‘node score cutoff = 0.2’, and ‘maximum depth = 100’. Hub genes were identified using the Cytoscape plugin cytoHubba by MCC method, as described previously^106^.

### Determining the putative miRNAs which target the viral and host components

Prediction of the human miRNAs that could target the viral RNA genome was performed using the miRDB custom search web tool^107^. This database allows for submission of the entire SARS-CoV-2 genome and provides the list of human miRNAs potentially targeting different regions of the viral genome. Prediction of the human miRNAs that target host genes interacting with the virus was performed using miRDB, miRanda, and TargetScan databases. Validated miRNA targets were obtained from miRTarBase and considered in combination with co-predicted gene targets for gene ontology and biological pathway analyses.

## Supplementary Information

Supplementary Information 1.

Supplementary Table S1.

Supplementary Table S2.

Supplementary Table S3.

Supplementary Table S4.

Supplementary Table S5.

Supplementary Table S6.

Supplementary Table S7.

Supplementary Table S8.

Supplementary Table S9.

Supplementary Table S10.
