# Supplementary material for: In silico analysis suggests the RNAi-enhancing antibiotic enoxacin as a potential inhibitor of SARS-CoV-2 infection
Source: Sci Rep. 2021 May 13;11:10271. doi: 10.1038/s41598-021-89605-6 (PMC8119475; doi:10.1038/s41598-021-89605-6)
Supplement: Supplementary file 1 — Supplementary Information 1. [file 41598_2021_89605_MOESM1_ESM.docx]

**Supplementary Information**

**In silico analysis suggests the RNAi-enhancing antibiotic enoxacin as a potential inhibitor of SARS-CoV-2 infection**

Amirhossein Ahmadi^1^, Sharif Moradi^2*^

1. Department of Biological Science and Technology, Faculty of Nano and Bioscience and Technology, Persian Gulf University, Bushehr, 75169, Iran
2. Department of Stem Cells and Developmental Biology, Cell Science Research Center, Royan Institute for Stem Cell Biology and Technology, ACECR, Tehran, Iran

***Correspondence:**

Sharif Moradi, Ph.D., Royan Institute, Banihashem Sq., Banihashem St., Ressalat Highway, Tehran, Iran. Postal Code: 1665659911, P.O. Box: 16635-148, Tel: +98 21 22306485, Fax: +98 21 23562507, Email: [sh.moradi@royan-rc.ac.ir](mailto:sh.moradi@royan-rc.ac.ir); [sharif.moradi@gmail.com](mailto:sharif.moradi@gmail.com)

ORCID ID: [http://orcid.org/0000-0002-2935-983X](https://www.scopus.com/redirect.uri?url=http://www.orcid.org/0000-0002-2935-983X&authorId=55849602800&origin=AuthorProfile&orcId=0000-0002-2935-983X&category=orcidLink)

This manuscript contains six supplementary figures and eight supplementary tables which can be found online at *Scientific Reports* website.

**Supplementary Figures**

**Figure S1.** The PPI network of host proteins potentially interacting with SARS-CoV-2 components together with the top four protein modules derived from the network.

**Figure S2.** Enrichment analysis of genes and the top modules which are predicted to interact with SARS-CoV-2 components.

**Figure S3.** Hub genes in the PPI network of proteins interacting with SARS-CoV-2 proteins and their pathway enrichment analysis.

**Figure S4.** The eight enoxacin-induced miRNA introduced by our study were previously reported to target SARS-CoV-2 genome.

**Figure S5.** Enoxacin-induced miRNAs might diminish the cytokine storm induced by SARS-CoV-2.

**Supplementary Tables**

**Table S1.** Predicted pri-miRNA structures in the SARS-CoV-2 genome.

**Table S2.** Enoxacin-induced miRNAs that are also expressed by the lung tissue.

**Table S3.** miRNA and mRNA profiles of lung tissue (extracted from IMOTA atlas).

**Table S4.** miRNAs potentially targeting *ACE*, *ACE2*, *TMPRSS2*, *CTSL*, *TMPRSS11D*, and *FURIN*.

**Table S5.** Union of predicted and validated genes targeted by enoxacin-induced miRNAs.

**Table S6.** Human proteins that interact with SARS-CoV-2 proteins (obtained from BioGRID database).

**Table S7.** 103 host proteins interacting with viral proteins could be targeted by enoxacin-induced miRNAs**.**

**Table S8.** miRNAs targeting the SARS-CoV-2 genome extracted by miRDB custom search tool.

**Table S9.** The list of enoxacin-induced miRNAs which might directly target the SARS-CoV-2 genome.

**Table S10.** miRNAs which target the developmental stage-specific transcription factors and marker genes of BASCs.

**Supplementary Figures**


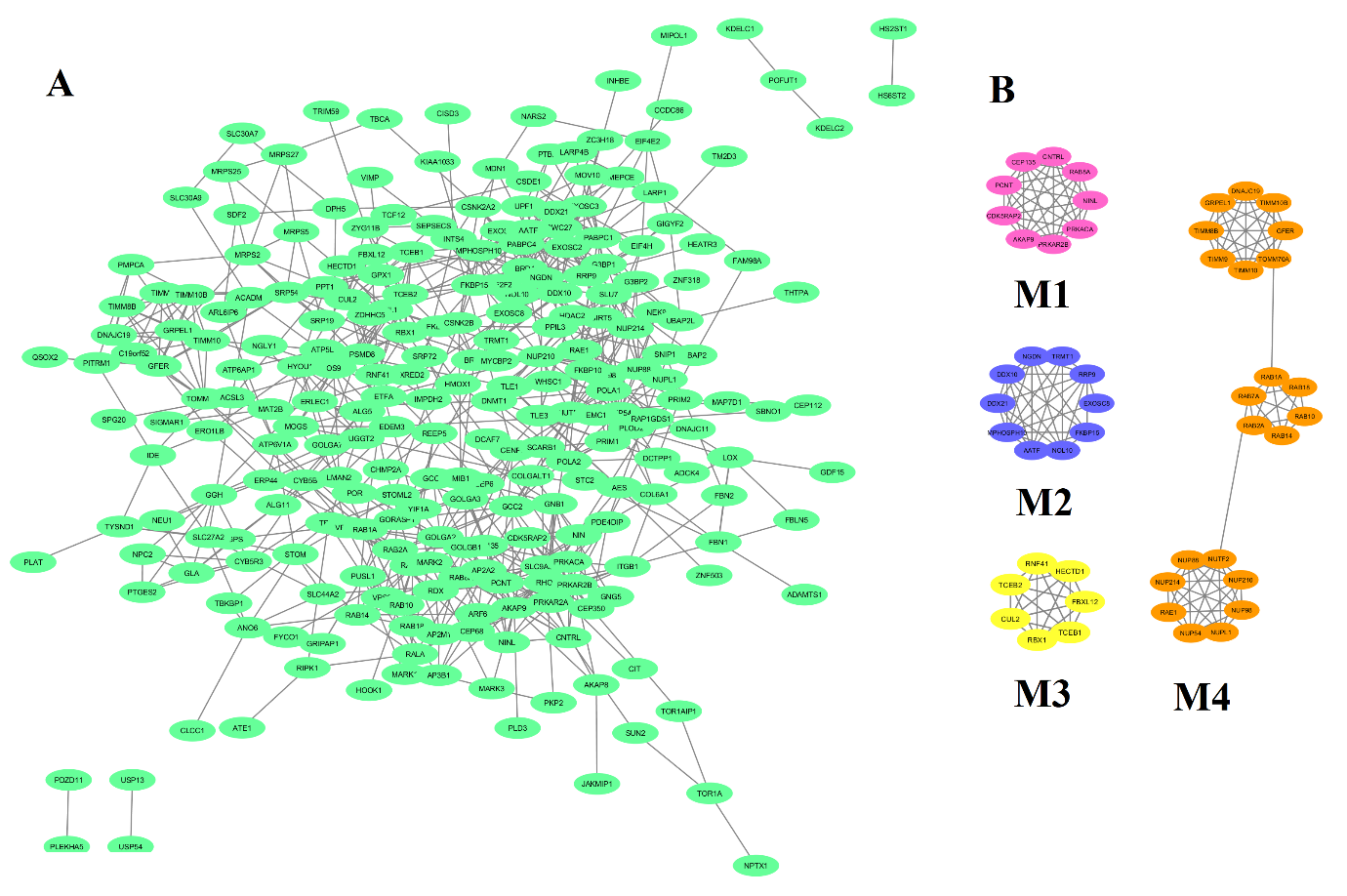


**Figure S1.** The PPI network of host proteins potentially interacting with SARS-CoV-2 components together with the top four protein modules derived from the network. (A) The PPI network of host proteins predicted to interact with SARS-CoV-2 components were depicted by Cytoscape (only interactions with the confidence of a combined score >0.400 were included). (B) The top four modules of the PPI network identified by MCODE (cutoff criteria were ‘degree cutoff=2’, ‘k-core=2’, ‘node score cutoff=0.2’, and ‘maximum depth=100) which are shown as distinct blocks outside of the PPI network. M: Module

**
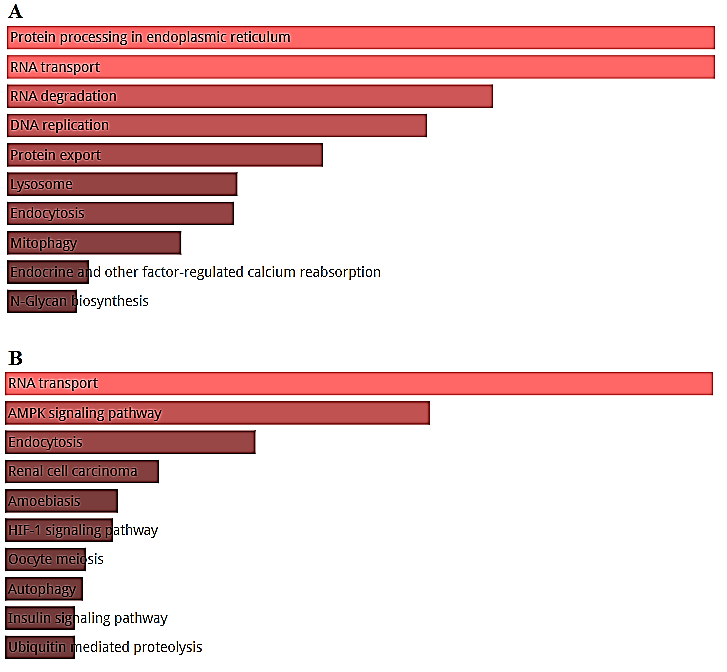
**

**Figure S2.** Enrichment analysis of genes and the top modules which are predicted to interact with SARS-CoV-2 components. (A) The KEGG enrichment analysis using Enrichr showed that proteins which interact with SARS-CoV-2 proteins, were mostly involved in protein processing in endoplasmic reticulum. (B) The KEGG pathway enrichment analysis revealed that the top four modules in the PPI network were mostly associated RNA transport. The lighter the red color is, the more significant the p-value.

**
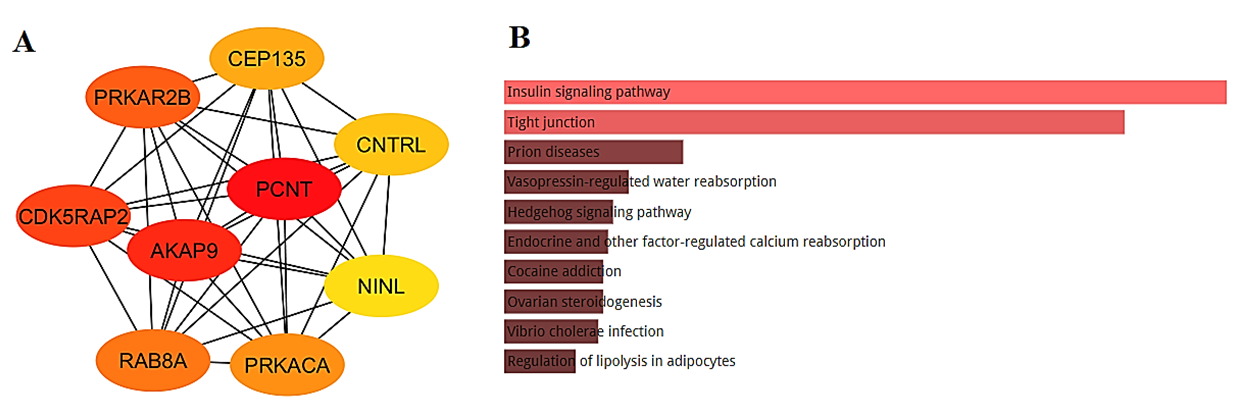
**

**Figure S3.** Hub genes in the PPI network of proteins interacting with SARS-CoV-2 proteins and their pathway enrichment analysis. (A) Nine hub genes were identified by Cytohubba and the MCC method. (B) The KEGG pathway enrichment analysis showed that these genes were mostly associated with insulin signaling pathway. The lighter the red color is, the more significant the p-value.

**
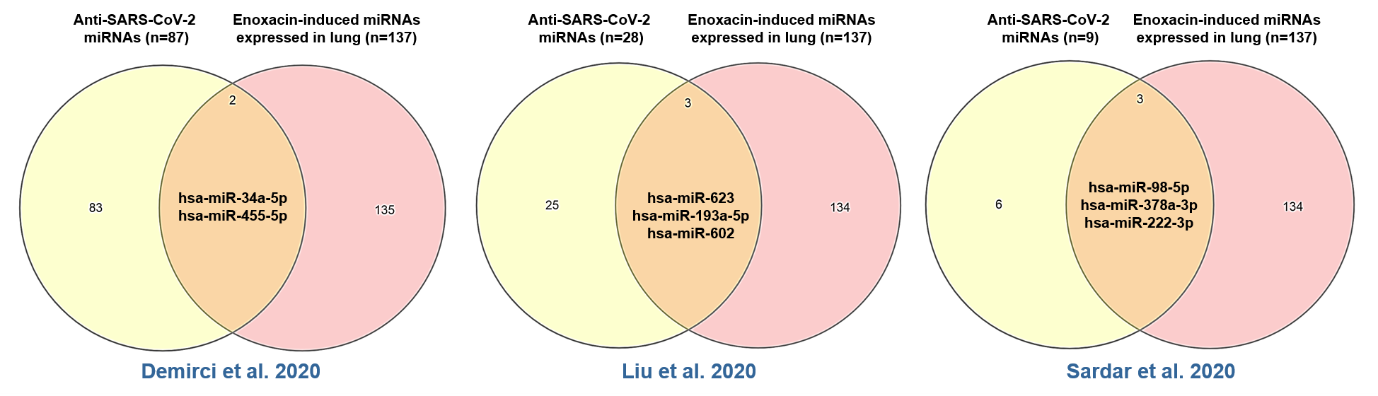
**

**Figure S4.** The eight enoxacin-induced miRNAs identified in our study have previously been reported to target the SARS-CoV-2 genome.

**
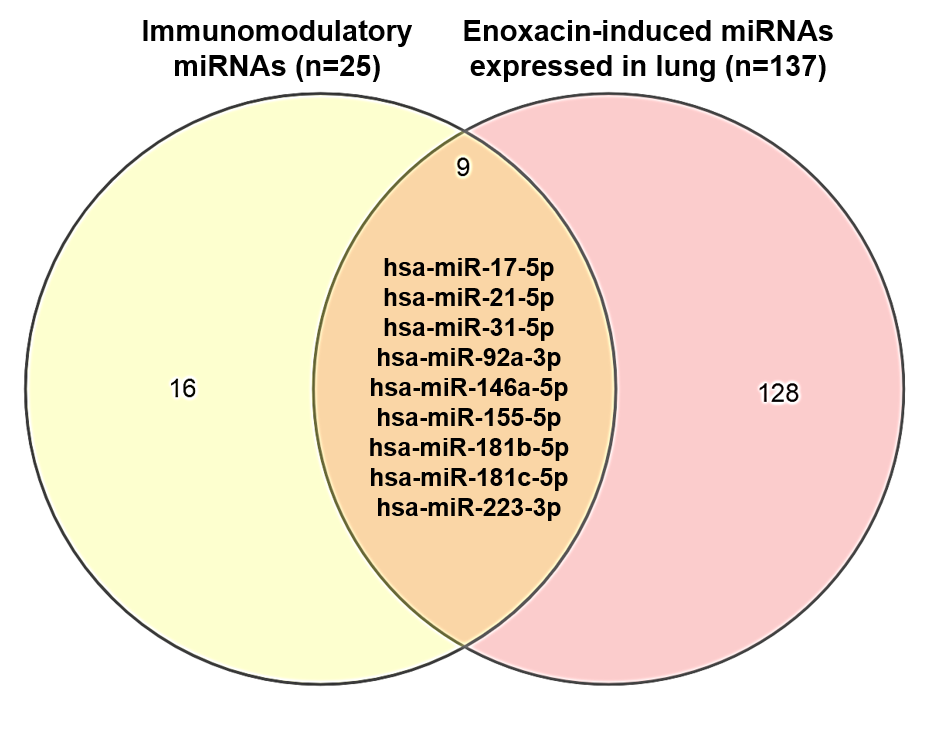
**

**Figure S5.** Enoxacin-induced miRNAs might diminish the cytokine storm induced by SARS-CoV-2. Several miRNAs with documented immunomodulatory roles are induced by enoxacin. In this Venn diagram analysis, we considered the enoxacin-induced miRNAs that were also expressed by the lung tissue. Notably, we observed no changes in the list of common miRNAs when considering all 268 enoxacin-upregulated miRNAs (data not shown).

**Supplementary Tables**

Supplementary tables can be found online at <https://www.nature.com/srep/>.
